# Maternal exposure to triclosan constitutes a yet unrecognized risk factor for autism spectrum disorders

**DOI:** 10.1038/s41422-019-0220-1

**Published:** 2019-08-28

**Authors:** Zijian Hao, Qionghui Wu, Zhengwei Li, Yali Li, Qiu Li, Xi Lai, Huan Liu, Menghuan Zhang, Ting Yang, Jie Chen, Yaping Tang, Jingkun Miao, Huatai Xu, Tingyu Li, Ronggui Hu

**Affiliations:** 1 University of Chinese Academy of Sciences; State Key Laboratory of Molecular Biology, CAS Center for Excellence in Molecular Cell Science, Institute of Biochemistry and Cell Biology, Chinese Academy of Sciences, Shanghai, 200031 China; 2 Pediatric Research Institute, Children’s Hospital of Chongqing Medical University, Chongqing Key Laboratory of Child Nutrition and Health, Chongqing, 400014 China; 3 Guangdong Provincial Key Laboratory of Brain Function and Disease, Zhongshan School of Medicine, Sun Yat-sen University, Guangzhou, Guangdong 510080 China; 4 Guangzhou Institute of Pediatrics, Guangzhou Women and Children’s Medical Center, Guangzhou Medical University, Guangzhou, Guangdong 510623 China; 5 Neonatal Screening Center, Chongqing Women and Children’s Medical Center, Chongqing, 401174 China; 6 Shanghai Institute of Neurosciences, Shanghai Institutes for Biological Sciences, Chinese Academy of Sciences, Shanghai, 200031 China

**Keywords:** Mechanisms of disease, Developmental biology

Dear Editor,

Autism spectrum disorders (ASD) are complex neurodevelopmental disorders characteristic of core behavioral traits like restricted, repetitive behaviors and deficiency in social interactions.^1^ The prevalence of ASD has rapidly increased worldwide, rising from 1/149 in 2000, 1/68 in 2012, and to ~1/40 in 2016 of children at the age of 3–17 in the United States.^2^ ASD prevalence was found to be associated with socioeconomic status (SES) as overpresented in high-income households,^3^ and to be significantly enriched in certain regions, e.g., reaching up to 4.88% in Florida.^2^ In the last decades, advances in genetic studies have led to unprecedented understanding of the genetic factors that were associated with < 50% of human ASD. Tens of thousands of genomic events or gene mutations were found in ASD patients. Among these genetic factors, many seemed to indeed play causal roles in ASD etiology as animals bearing some of ASD-associated mutations manifested autistic-like behaviors.^4–7^ Since genetic factors are usually SES independent and unlikely to change in every few years, non-genetic factors including changes in life styles, environmental factors and their complex interaction with genetic factors are believed to prominently contribute to the recent rapidly increasing ASD prevalence.^4–6^ However, their nature and mechanisms of action remain largely undetermined.

Triclosan (5-chloro-2-(2,4-dichlorophenoxy)phenol: TCS, Fig. 1a) is an antimicrobial agent pervasively used as a key ingredient in over 2000 healthcare, cosmetics or personal care products (such as hand washes, toothpastes, liquid soaps, creams and dinnerware detergents).^8^ TCS is also widely employed as preservative in food or products such as kitchen utensils and furniture, which could be particularly important to prevent the proliferation of microbes in tropical and stormy states like Florida. TCS goes into water and soils, and significantly accumulates within food chains. By penetrating human skin or being swallowed through mouth and digestive duct, TCS can enter human body efficiently and circulate in body fluids, remain in plasma and breast milk, and reach up to ~3000 µg/L in urines of high-income individuals.^9^ Therefore, the bioaccumulation of TCS exhibits a SES-dependent pattern, similar to that of ASD.^3^ Despite multiple alerting safety concerns,^9^ the rising demand for better hygiene is to drive fast expansion of TCS consumption worldwide.

**Fig. 1:**
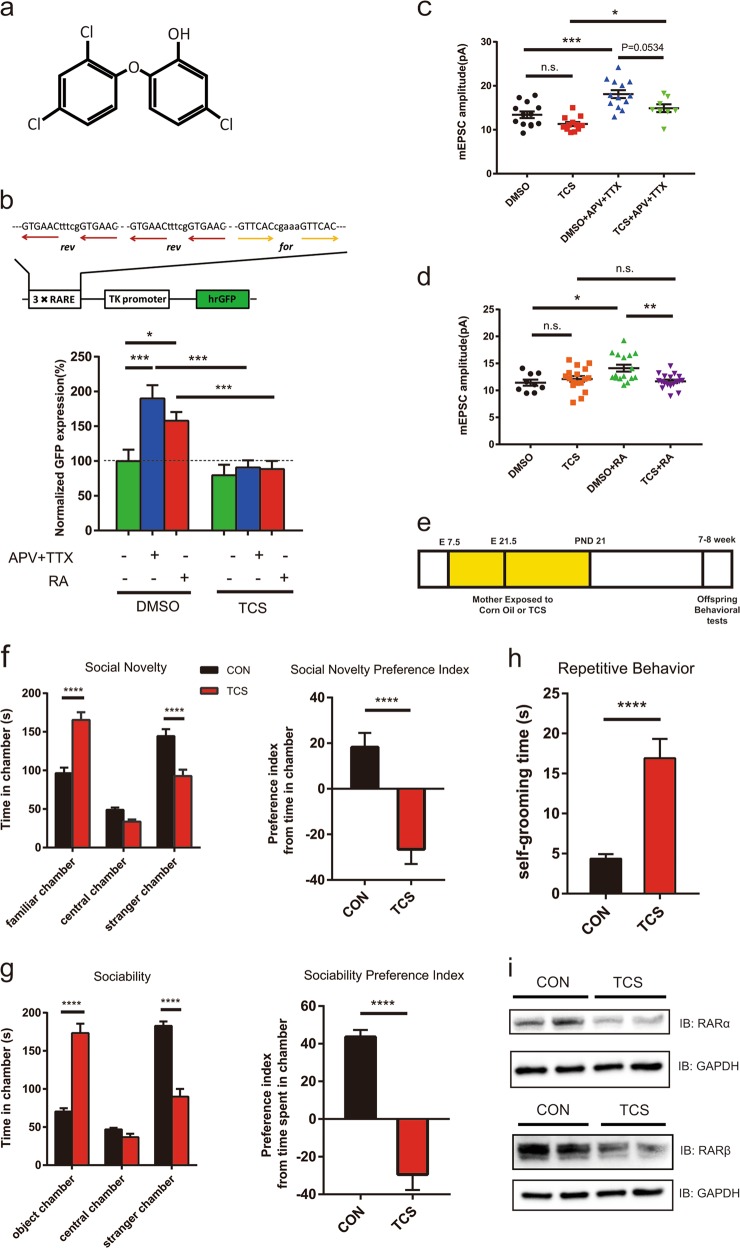
TCS downregulates RA signaling, disrupts synaptic plasticity and induces autistic behaviors in offspring rats. **a** Chemical structure of TCS. **b** Concurrent treatment with TCS (12.50 µM) for 12 h abolished the increase of RARE-hrGFP reporter expression (*n* = 22, 25, 22). The expression of hrGFP is driven by three copies of RARE upstream of TK promoter. Neuronal activity blockade by APV (100 µM) and TTX (1 µM) treatment for 24 h, or acute RA (10 µM) treatment for 8 h, significantly increased the expression of RARE-hrGFP (*n* = 25, 27, 23), but had no effect on the control reporter that did not have RARE (Supplementary information, Fig. S2). The mean values of normalized GFP expression were 100.00, 189.89, 157.81, 79.54, 90.77, 88.39, respectively. **c**, **d** Treatment of APV and TTX or RA (1 µM) significantly induced synaptic upscaling. Addition of TCS disrupted the synaptic scaling, manifested as reduced mEPSC amplitudes (**c**, *n* = 13, 12, 13, 8; mean values of amplitude were 13.40, 11.30, 18.12, 14.89, respectively; **d**, *n* = 9, 17, 16, 18; mean values were 11.42, 12.12, 14.12, 11.67, respectively). Representative mEPSC traces of PFC neurons were shown in Supplementary information, Fig. S4. **e** A scheme for TCS administration. Starting from E7.5, wild-type pregnant Sprague-Dawley rats were orally administered with TCS (50 mg/kg/d) until weaning at PND21. Offspring rats were subjected to behavioral tests at the age of 7–8 weeks. **f**–**h** Pups from mother rats exposed to TCS manifested autistic-like behaviors. They tended to spend more time with familiar subject in social novelty tests (**f**), more time with object in sociability tests (**g**), and longer time on self-grooming (**h**), compared with pups in control group (CON). CON group, *n* = 38; 50 mg/kg/d TCS group, *n* = 39. **i** Representative western blots of RARα and RARβ proteins using lyzed PFC tissues from two offspring rats of the control or TCS group. Data are means ± SEM. *P *<* 0.05, **P < 0.01, ***P *<* 0.001, ****P *<* 0.0001, n.s., not significant. One-way ANOVA with Bonferroni *post hoc* test was used to analyze fluorescence assay and electrophysiology results. Self-grooming time and preference index were analyzed by unpaired two-tailed Student’s *t*-test with Welch’s correction. For three-chamber experiments, two-way repeated-measures analysis of variance (ANOVA) was used. Detailed statistical analysis results were presented in Supplementary information, Tables S1 and S2

Retinoic acid (RA) is both a morphogen and signaling molecule with prominent roles in mediating neuron differentiation, synaptic plasticity and tissue formation. Recently, we reported that ASD-like behaviors could be induced in mice upon downregulation of cellular RA synthesis, through mimicking ASD-associated UBE3A overdosage or chemical inhibition of RA synthesis with disulfiram, and such ASD behaviors could be efficiently reversed by oral supplementation of RA into the male mice.^10^ This has established a potentially causal role of down-regulated RA signaling in ASD etiology. Later, fragile X mutations were also found to undermine RA signaling,^11^ and some ASD patients bore mutations in RA receptor A (*RARA*), which disrupted downstream RA signaling.^12^

Homeostatic synapse plasticity plays an important role in maintaining the stability of neuronal circuits. Neuronal RA synthesis has been shown to be induced and required by synaptic scaling, as one type of homeostatic plasticity, upon activity blockade with the sodium channel blocker tetrodotoxin (TTX, 1 μM) and the NMDA receptor antagonist D(−)-2-Amino-5-phosphonopentanoic acid (D-APV, 100 μM) in neurons.^13^ As shown in Fig. 1b, the treatment with both APV and TTX or RA alone could increase the expression of humanized *Renilla reniformis* green fluorescent protein (hrGFP) driven by RA-responsive element (RARE), which could be efficiently blocked by addition of TCS (also Supplementary information, Fig. S1). However, the same treatment did not have a significant effect on the expression of the control reporter driven by constitutive TK (Thymidine Kinase) promoter (Supplementary information, Fig. S2), suggesting a RARE-specific inhibitory effect of TCS on gene expression. Indeed, co-treatment with APV and TTX elicited synaptic upscaling, as manifested by the marked increase (from 13.40 to 18.12 pA) in miniature excitatory postsynaptic current (mEPSC) amplitude in rat prefrontal cortex (PFC) neurons. Such an increase in mEPSC amplitude could be potently suppressed by treatment of 12.50 μM TCS, which was lowered to an average 14.89 pA (Fig. 1c). Notably, TCS treatment did not lead to a significant change in mEPSC amplitude, when compared to that recorded for the DMSO group (Fig. 1c, d). As an indication of RA-inducible synaptic upscaling in PFC neurons, the addition of RA alone was also able to induce an increase in mEPSC amplitude from 11.42 pA to 14.12 pA. However, in the presence of 12.50 μM TCS, the mEPSC amplitude did not significantly change after RA treatment, with mEPSC amplitude recorded as ~11.67 pA. Since addition of RA did not induce increase in mEPSC amplitudes when TCS was present, it was likely that TCS disrupted RA signaling or RA-mediated synaptic upscaling upon activity blockade through mechanisms involving events downstream of RA synthesis (Fig. 1b, d). As reported before, none of the above treatment seemed to affect mEPSC frequency in the same neurons (Supplementary information, Figs. S3 and 4). Taken together, these findings clearly indicated that TCS, once entering brain, could directly disrupt RA-induced gene expression and suppress RA-mediated synaptic upscaling in neurons.

Previously, the oral ‘No Observed Adverse Effect Level’ (NOAEL) of TCS for rat was found to be 50 mg/kg/d, as TCS at this dose seemed to have no or little adverse effects on the health of dams and pups.^14^ Wild-type pregnant rats were then subjected to oral gavage with 50.0 mg/kg/d TCS (dissolved in corn oil) from day 7.5 of pregnancy (E7.5) until weaning (postnatal day 21, PND21), and the pups of both genders were then subjected to behavior tests 7–8 weeks after birth (Fig. 1e). No significant differences were detected in body weights of dams and offspring between the control and TCS-exposed groups (Supplementary information, Fig. S5). Compared to the control group whose mothers received corn oil only, the pups maternally exposed to TCS were found to spend a longer time in chambers containing familiar rats or objects but a significantly less time with the stranger rats (Fig. 1f, g). The offspring from the TCS-fed mother rats also spent a longer time in self-grooming than those from control group (Fig. 1h). The time spent in central zone of open field for TCS-exposed rat pups was markedly lower than that for control pups, indicating an increased anxiety, while the motility of pups in both groups was comparable (Supplementary information, Fig. S6). Furthermore, as shown by immunoblotting analysis using lyzed PFC tissues (Fig. 1i), the expression of endogenous *Rara* (retinoic acid receptor alpha) and *Rarb* (retinoic acid receptor beta), both target genes and mediators of RA signaling, was significantly lower in the rat brains of the TCS group in comparison to that of the control group, suggesting that maternal exposure to TCS disrupted at least part of the neuronal RA signaling in the offspring brains. This result also indicated the impairment of RA signaling in PFC after exposure to TCS. Taken together, these data suggest that maternal exposure to TCS might cause autistic-like behaviors in the offspring, most likely through disrupting the expression of the target genes of RA signaling in brain.

Since ASD showed a male bias in prevalence with a ratio of 4:1 (male:female) in humans, it was intriguing to ask whether there exists gender-specific behavioral traits in the offspring maternally exposed to TCS. The data from the above behavior tests were re-analyzed for gender specificity. It was evident that the maternal exposure to TCS did not cause gender-specific behavioral traits in the offspring rats (Supplementary information, Fig. S7).

In addition to genetic factors, so far many potential risk factors such as paternal aging, nutrition, pollution and other endocrine-disrupting chemicals, have been suggested to contribute to the etiology of human ASD. Among them, the increasingly accepted concept of DoHaD (Developmental Origins of Health and Disease) highlights potential avenues and mechanisms through which exposure to certain environmental factors during early development of an individual may cause an expanding set of human diseases.^15^ Our work presented here strongly suggested that disrupting RA signaling by commonly used chemicals like TCS might represent an important, yet under-explored, DoHaD mechanism through which an expanding spectrum of human disorders originates, e.g., ASD. Obviously, differential exposures to such chemicals together with the heterogeneous genetic compositions in humans, which determine the metabolic rates and cellular responses to these chemicals, may at least partially underlie the currently observed group-specific pattern of the human diseases, i.e., SES-dependent and geologically enriched distribution in ASD prevalence. Identification of the potential threats that these agents pose to our health may constitute the first critical step for us to prevent the related maladies. Notably, since TCS acts primarily as an antimicrobial agent, it is possible that TCS might induce autistic behaviors through mechanism besides downregulation of cellular RA signaling, e.g., altering the composition of gut microbiome in the mother rats and the offspring as well. Indeed, there are projects in our lab ongoing to profile TCS-induced changes in the spectrum of gut microbiota in both the TCS-fed mother rats and their offspring to further pinpoint specific factors that may also contribute to the above observed TCS-induced behavior traits. Further studies are warranted to determine whether and how the maternal exposure to TCS may potentiate the risk of ASD in humans.

The use of TCS has been generally believed to be safe, as the oral LD50 (lethal dose, 50%) for mice was determined as 4530 mg/kg. Despite the fact that Food and Drug Administration of the United States of America banned the use of TCS among 19 active ingredients used in over-the-counter (OTC) consumer antiseptic products, TCS is still included in over 2000 types of products, which is well poised to further significant increase in the near future. Meanwhile, although the dose for daily TCS exposure seemed to be low among general population, TCS could significantly accumulate in human body due to partial excretion and slow turnover, reaching up to 3.79 mg/L in urines.^9^ Our findings presented above have, unexpectedly, revealed a yet unrecognized impact of TCS on behavioral traits of mammalian pups, thus establishing it as a novel environmental factor that could significantly contribute to the etiology of ASD. In the current practice of safety assessment for chemicals, attentions are usually only paid to the physiological health of animals subjected to the agents, with little or no effort taken to examine their potential impact on the mental health of animals, let alone their offspring. To better safeguard the populace from unwanted consequences, it is the right time to re-evaluate whether and how many other routinely used chemicals might impact the mental health of animals and humans, and act accordingly.

## Supplementary information

Supplementary information
