# Supplementary material for: Maternal exposure to triclosan constitutes a yet unrecognized risk factor for autism spectrum disorders
Source: Cell Res. 2019 Aug 28;29(10):866–9. doi: 10.1038/s41422-019-0220-1 (PMC6796921; doi:10.1038/s41422-019-0220-1)
Supplement: Supplementary file 1 — Supplementary information [file 41422_2019_220_MOESM1_ESM.pdf]

Supplementary information

Materials and Methods

Drugs

Corn oil and Tetrodotoxin (TTX) was purchased from Aladdin Chemicals Inc. D-APV was purchased from Tocris Bioscience, UK. Triclosan (TCS) used for neuron treatment was purchased from Target Molecule Corp (TargetMol, USA). Triclosan used for oral administration and Dimethyl Sulfoxide (DMSO) was purchased from Sigma Aldrich, USA. All-trans retinoic acid (ATRA, also named as Tretinoin) was purchased from Dr. Ehrenstorfer GmbH, Germany.

Cell culture, transfection and drug treatment in fluorescence assay

Primary rat prefrontal cortex (PFC) neurons were freshly prepared from the brains of wild-type Sprague-Dawley rats on embryonic day 17, cultured with DMEM/F-12 (Gibco, 11330032) medium supplemented with 10% FBS on 0 day in vitro (DIV) and switched to serum-free Neurobasal medium (Gibco, 21103049) supplemented with B-27 (Gibco, 17504044) and GlutaMAX (Gibco, 35050061) on the next day. At 8 DIV, primary rat PFC neurons were transfected with indicated reporter plasmids using Calcium Phosphate Cell Transfection Kit (Beyotime, C0508), 24 h prior to chemical treatments. The neurons were then either treated with 1.0 μM TTX and 100.0 μM D-APV for 24 h and DMSO or DMSO-dissolved 12.5 μM TCS for 12 h, or treated with DMSO or 12.5 μM TCS for 12 h and 10.0 μM RA for 8 h.

Fluorescence assay and image acquisition

The plasmid containing pCDH-RARE-TK-hrGFP reporter was constructed as previously

described [1]. Briefly, three copies of RA response element (RARE) were placed upstream of TK promoter, which drives the expression of humanized *Renilla reniformis* green fluorescent protein (hrGFP). Twenty-four hours after the transfection at 8 DIV, the PFC neurons were treated with indicated drugs before they were fixed with 4.0% paraformaldehyde for 20 min at room temperature and washed with phosphate buffer saline (PBS) 3 times before mounting. Fluorescent images were acquired at room temperature with Leica TCS SP8 confocal microscope, using a 63× oil objective (N.A. 1.40, WD 0.17), in sequential acquisition setting at 1024 × 1024 pixel resolutions. Identical settings for laser power, gain, and offset were used in the same experiments. At least 20 neurons from two culture wells were analyzed per condition in one experiment, and at least two independent experiments were performed. Fluorescence intensity was analyzed by ImageJ Fiji software.

Electrophysiology

As described before [1], whole-cell patch-clamp recordings of neurons at 12-15 DIV were measured at room temperature after the treatment with indicated drugs. Specifically, neurons were treated with DMSO or 12.5 μM TCS alone for 24 h, or along with 1.0 μM TTX and 100.0 μM D-APV for 24 h, or along with 1.0 μM RA for 45 min. Patch recording pipettes (8-12 MΩ) were filled with 20.0 mM HEPES, 5.0 mM $MgCl_2$, 110.0 mM K-gluconate, 20.0 mM KCl, 0.2 mM Na_3-GTP, 2.0 mM Na_2-ATP, 0.6 mM EGTA (pH 7.3, 290.0 mOsm) as the internal solution. The external solution (25.0 mM HEPES, 1.0 mM $MgCl_2$, 30.0 mM glucose, 5.0 mM KCl, 2.0 mM $CaCl_2$, 129.0 mM NaCl, pH 7.3, 310.0 mOsm) was used to maintain cell cultures. 1.0 μM TTX and 100.0 μM picrotoxin (Tocris) were added to the external solution for mEPSC recordings. Cells were held at –70 mV. Events from the neurons were recorded and analyzed on Mini-Analysis System (Synaptosoft).

Animals

Sprague-Dawley (SD) rats were housed in standard animal cages under specified pathogen-free (SPF) laboratory conditions in the animal facility at the Children's Hospital of Chongqing Medical University, experimentally manipulated with the approval by institute committee for animal welfares of Shanghai Institute of Biological Sciences IBS and Chongqing Children's Hospital. All the female rats were housed in groups (n = 3-5) before they were impregnated.

Maternal exposure to TCS

The SD rats were mated overnight and females were examined every morning for the presence of vaginal plugs, noted as gestation day 0.5 (GD0.5). Body weights for the pregnant rats were recorded from GD7.5 every day before administration until postnatal day 21 (PND21). TCS was freshly dissolved in corn oil at 50.0 mg/mL (m/v). Pregnant rats were administered with a single dose of TCS (50.0 mg/kg) every day via oral gavage since GD7.5 until weaning (PND21), while the control group received corn oil only. Pups from both control and TCS groups were weighted every week for 8 weeks, and subjected to behavioral tests at the age of 7 weeks, recorded through the ANY-Maze Video Tracking System (ANY-Maze, USA).

Animal behavior tests

Animal behavior tests were performed according to standard procedures described before [1-3] with slight modifications. The details were presented below.

1) Self-grooming test

The open field arena consisting of a dark opaque acrylic cube (48.0 × 48.0 × 60.0 cm^3) was divided into a center zone (24.0 × 24.0 cm^2) and a peripheral zone outside the central area. The test rats were placed in a fixed corner of the open field and facing the corner. Each test rat was observed for 5 min in the open field. Time spent in the center zone was automatically tracked

by the ANY-Maze Video Tracking System and total self-grooming time was recorded by trained personnel who were kept unaware of the group assignment.

2) Three-chamber tests

The three-chamber social tests were performed in an open-top transparent acrylic box (60.0 × 30.0 cm^2) that was equally divided into three chambers (each 20.0 × 30.0 cm^2) with holes on walls providing passage between the chambers. Each test rat was introduced to the central chamber and allowed to explore the three chambers freely for 5 min for habituation before the day of formal test. The three-chamber test was performed for three days including the habituated day, with all test animals housed in the test room.

In the first stage-sociability test, while a static toy and an unfamiliar sex- and age-matched rat (the stranger) were placed in left and right cages, respectively, the middle chamber was left empty until the test animal was introduced. The time to perform test in the apparatus was set to 5 min, with the time spent in each chamber was automatically recorded and later calculated using the ANY-Maze Video Tracking System.

For each test animal, 24 h after the sociability test, the social novelty test was started by having an unfamiliar sex- and age-matched rat as the stranger, held in a clear wire cage and placed in the right chamber, while a familiar sex- and age-matched rat as the familiar was held in a clear wire cage and kept in the left chamber. The time that the test animals spent in each chamber was recorded and later calculated on the ANY-Maze Video Tracking System.

Western blotting of proteins in PFC region

Prefrontal cortex from two pups of each treatment group was collected. The PFC extracts were weighted for 50 mg and was homogenized with 500 µL ice-cold RIPA buffer (KeyGEN BioTECH, KGP702-100) with protease inhibitors (KeyGEN BioTECH, KGP603), and was

incubated on ice for 30 min. After centrifugation at 10,000 r/min, 4 °C for 10 min, the protein in supernatant was quantified by BCA Protein Assay Kit (ATGene, ATP-0026-1). The equal amount of protein was added with loading buffer and boiled for 10 min. Western blotting experiments were performed with anti-RARA (1:1000, GeneTex, GTX54703), anti-RARB (1:1000, Abcam, ab53161) and anti-GAPDH (1:5000, Proteintech, HRP-60004).

Supplementary References

1. Xu X, Li C, Gao X, *et al.* Excessive UBE3A dosage impairs retinoic acid signaling and synaptic plasticity in autism spectrum disorders. *Cell Research* 2018; **28**:48–68.

2. Nishitani N, Nagayasu K, Asaoka N, *et al.* Manipulation of dorsal raphe serotonergic neurons modulates active coping to inescapable stress and anxiety-related behaviors in mice and rats. *Neuropsychopharmacology* 2018:1.

3. Lai X, Wu X, Hou N, *et al.* Vitamin A Deficiency Induces Autistic-Like Behaviors in Rats by Regulating the RARβ-CD38-Oxytocin Axis in the Hypothalamus. *Molecular Nutrition & Food Research* 2018; **62**:1700754.

Supplementary information, Figure S1

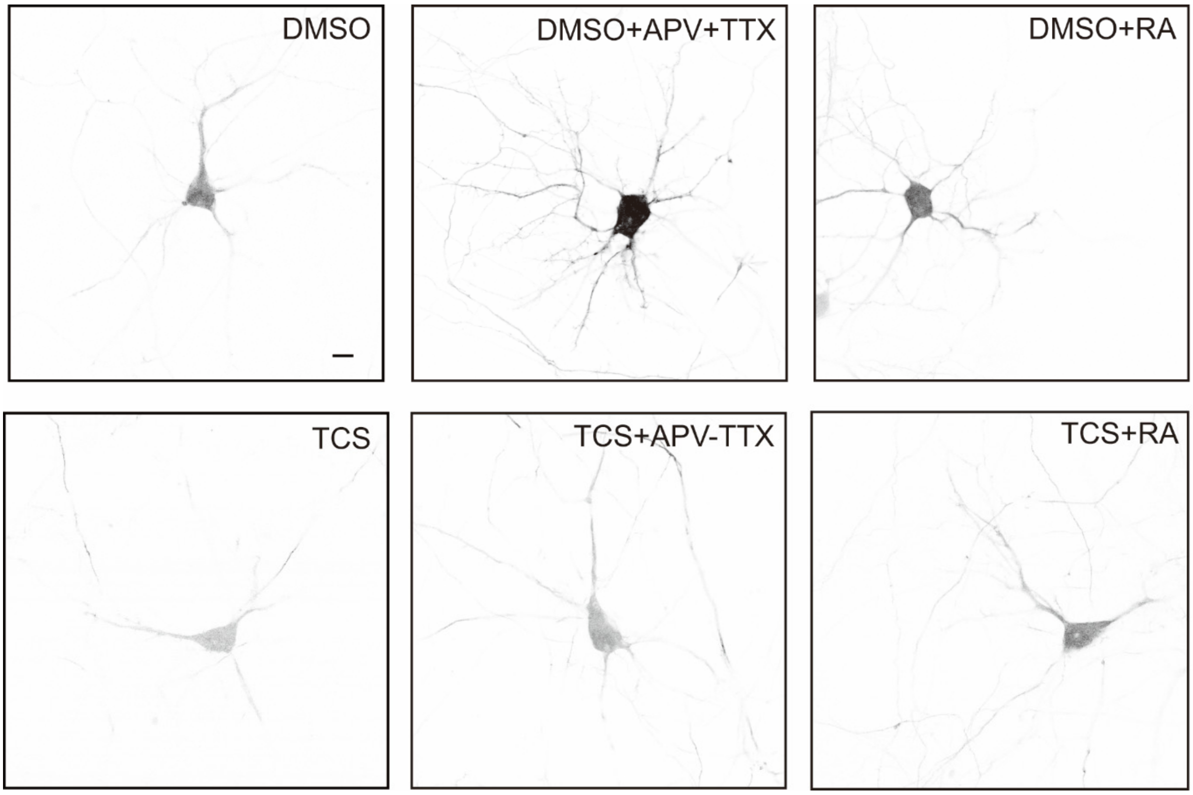

Fig. S1 Representative images of 3×RARE-TK-hrGFP reporter expression in rat PFC neurons under the treatment of indicated drugs. Scale bar: 10 μm.

Supplementary information, Figure S2

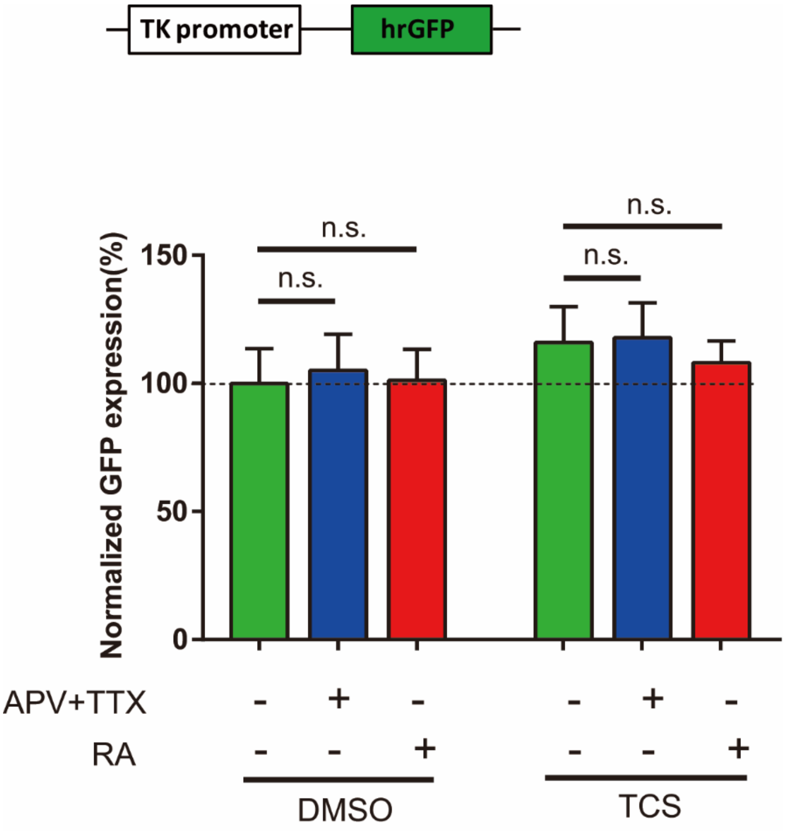

Fig. S2 Expression of control hrGFP reporter was not significantly changed by the treatment of indicated drugs. Schematics of the control hrGFP reporter (without RARE). Treatment of APV (100 μM) and TTX (1 μM) or RA (10 μM) or TCS (12.5 μM) did not significantly change the expression of control hrGFP reporter. For DMSO group, n = 22, 19, 24. For TCS group, n = 22, 21, 23. Data are presented as means ± SEM; n.s. not significant, $P > 0.99$; One-way ANOVA with Bonferroni *post hoc* test.

Supplementary information, Figure S3

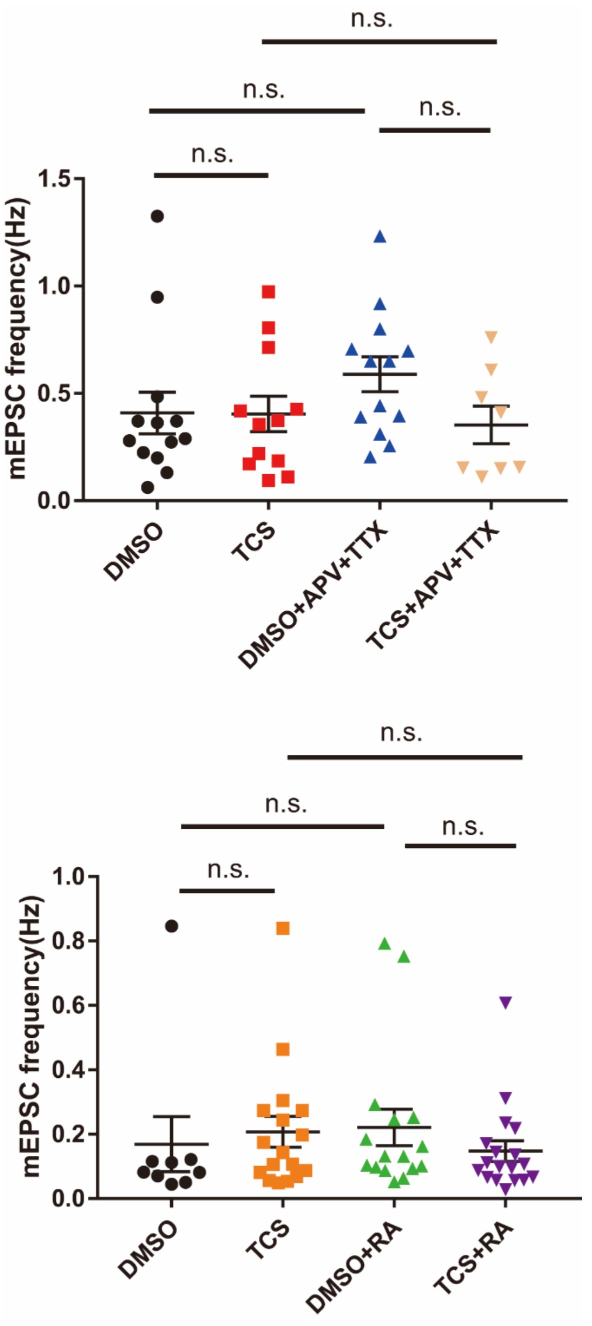

Fig. S3 Treatment of APV+TTX or RA or TCS did not significantly change the frequency of mEPSC in rat PFC neurons. Treatment of APV (100 μM) and TTX (1 μM) for 24 h, or RA (1 μM) for 1 h, or TCS (12.5 μM) for 24 h, did not significantly change the mEPSC frequency (APV+TTX panel, *n* = 13, 12, 13, 8; RA panel, *n* = 9, 17, 16, 18). Data are presented as means ± SEM; n.s. not significant, $P > 0.29$; One-way ANOVA with Bonferroni *post hoc* test.

Supplementary information, Figure S4

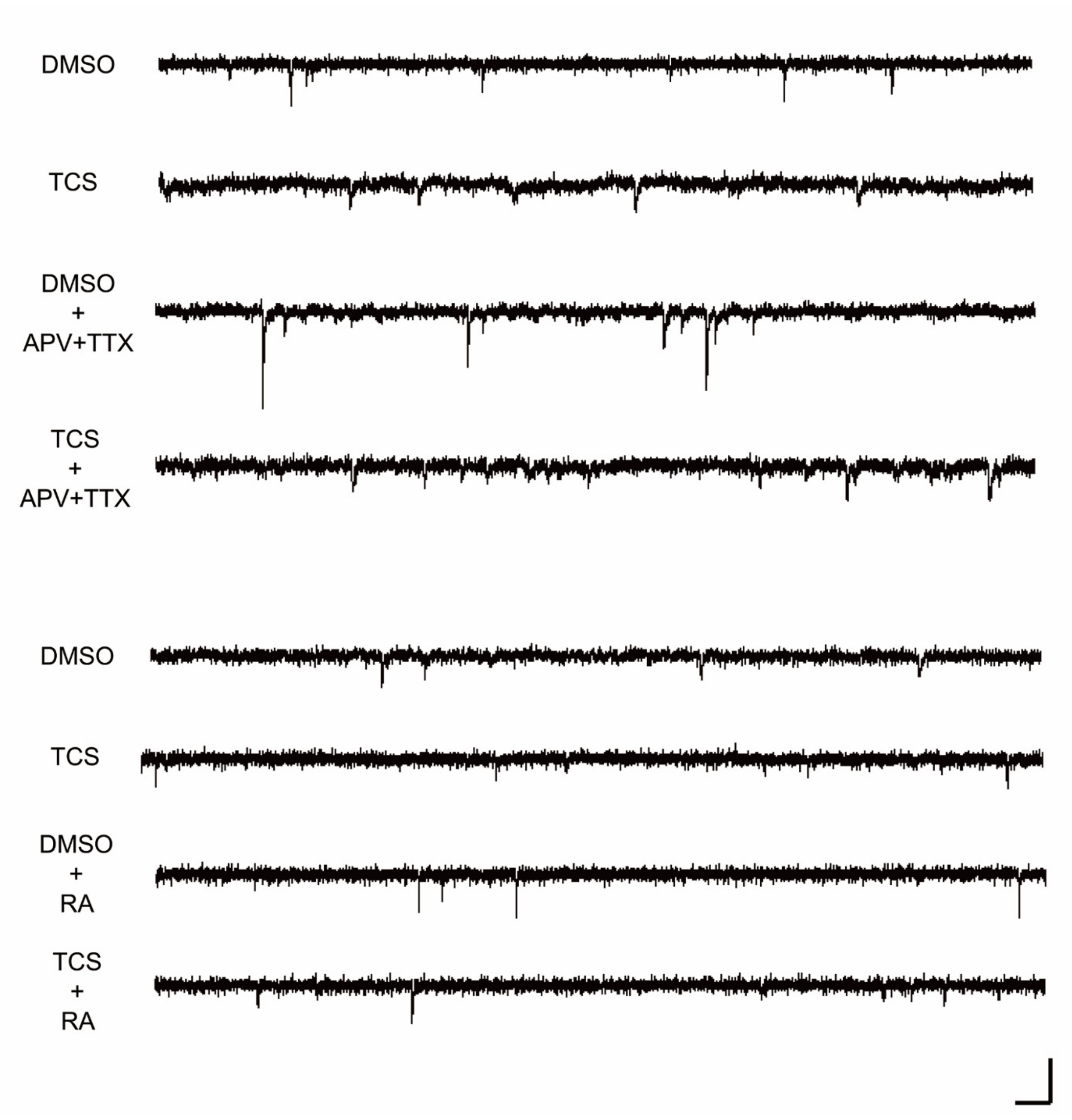

Fig. S4 Representative mEPSC traces of rat PFC neurons under the treatment of indicated drugs. Scale bar: 20 pA, 100 ms.

Supplementary information, Figure S5

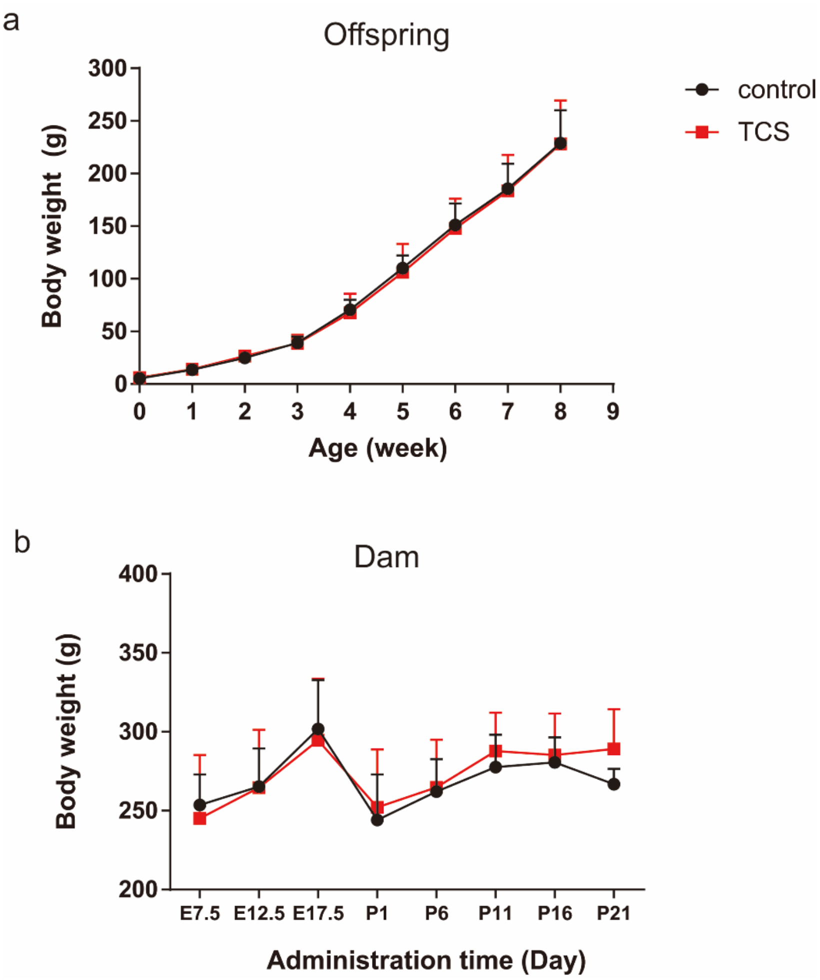

Fig. S5 Administration of 50 mg/kg/d TCS on maternal rats did not cause significant differences in body weights of offspring or dams, compared to those of control group. **a** Offspring were weighted every week after birth until the behavioral tests were finished. No significant weight difference was observed between the two groups. For control group, n = 38. For TCS group, n = 39. **b** Body weights of dams were recorded every day from embryonic day 7.5 (E7.5) till postnatal day 21 (P21), throughout the administration period. The body weights on E7.5, E12.5, E17.5, P1, P6, P11, P16 and P21 were shown on the graph. n = 4 for each group. Data are presented as means ± SD. Two-way ANOVA with Bonferroni *post hoc* test.

Supplementary information, Figure S6

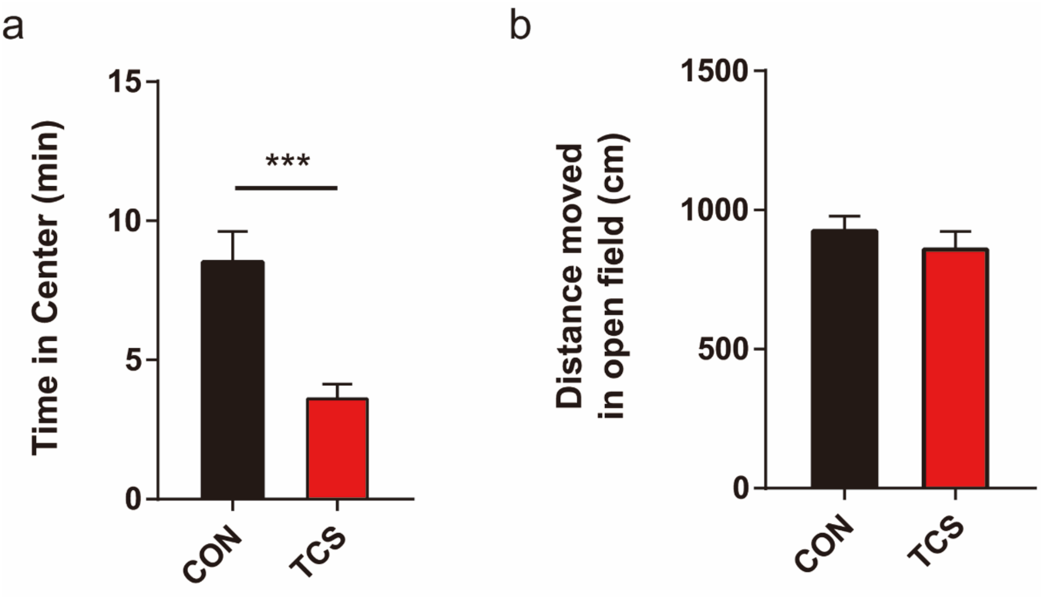

Fig. S6 Offspring rats from mothers exposed to TCS showed increased anxiety and comparable motility, compared to those from control group. **a** Time spent in central area in the open field test for offspring rats from TCS-treated mothers and control mothers. Data are means ± SEM. Unpaired two-tailed Student's *t*-test with Welch's correction. ***$P < 0.001$ (CON, $n = 38$; TCS, $n = 39$). **b** Total distance moved in the open field test for offspring rats from control group ($n = 38$) and TCS group ($n = 39$). Data are means ± SEM. Unpaired two-tailed Student's *t*-test with Welch's correction.

Supplementary information, Figure S7

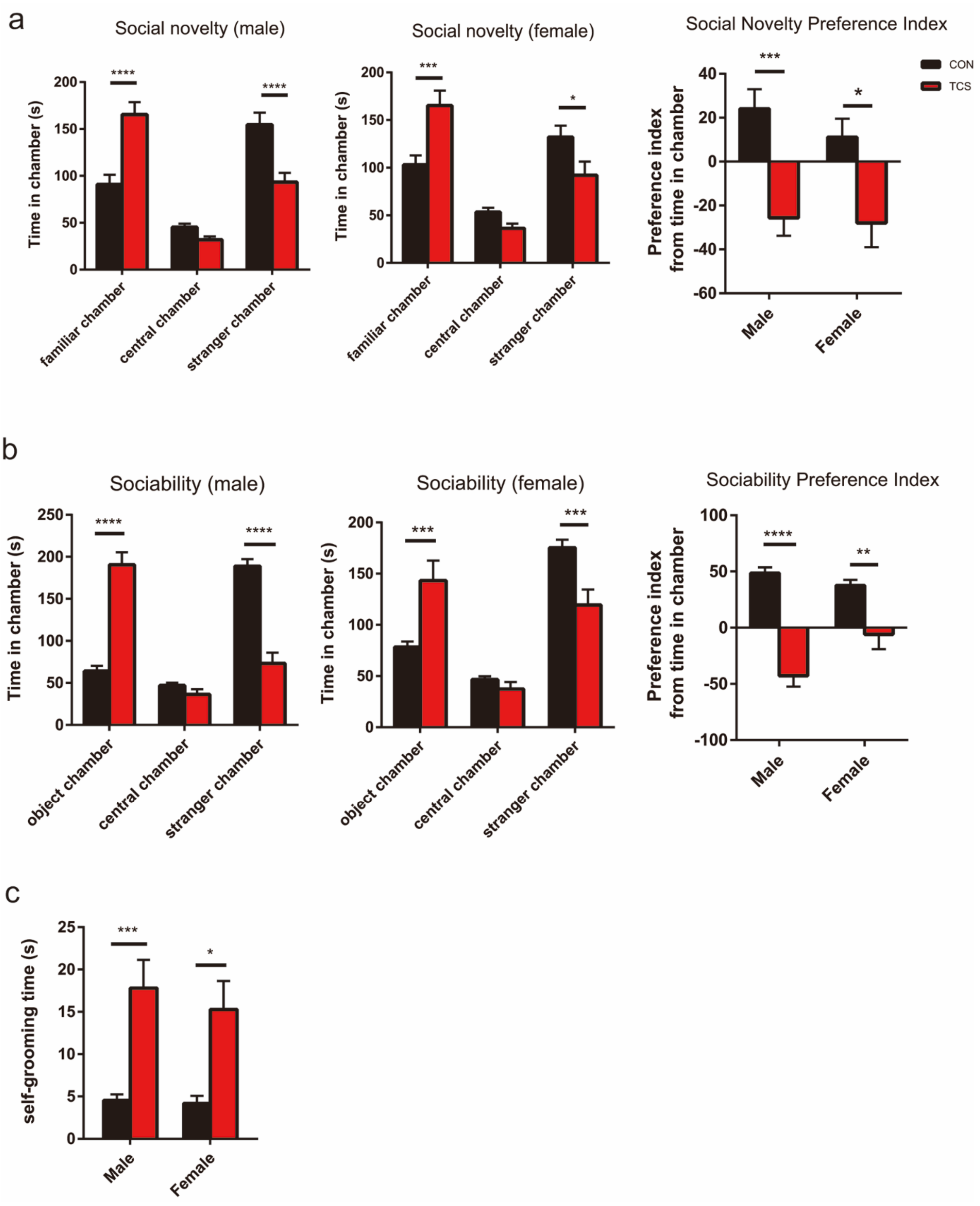

Fig. S7 Offspring rats of both genders manifested autistic-like behaviors in three-chamber sociability and social novelty tests and self-grooming test. No significant difference was

observed between male and female rats. **a, b** Time spent in chambers containing familiar rats and stranger rats **(a)**, or objects and living rats **(b)**, for male or female littermates from control group and TCS group. Preference index was also calculated. Data are means ± SEM. Two-way ANOVA with Bonferroni *post hoc* test. **c** Time spent in self-grooming for offspring rats was recorded and compared between control group and TCS group. Data are means ± SEM. Unpaired two-tailed Student's *t*-test with Welch's correction. $*P < 0.05$, $**P < 0.01$, $***P < 0.001$, $****P < 0.0001$. A total of 38 control group offspring rats (21 male and 17 female) and 39 TCS group offspring rats (25 male and 14 female) were subjected to behavioral tests.

Supplementary information, Table S1

Table S1 Chamber time (in seconds) and self-grooming time for Fig. 1f, g, h

Corresponding to Fig. 1f

CON vs TCS

	"Familiar" (s)		"Central" (s)		"Stranger" (s)	
	Mean	SEM	Mean	SEM	Mean	SEM
CON	96.38	7.14	48.91	2.99	144.50	9.00
TCS	165.42	9.98	33.59	2.79	92.77	8.17

	Mean (s)	SEM (s)
CON	18.25	6.244
TCS	-26.49	6.472
95% confidence interval	-62.66 to -26.83	
Welch-corrected t, df	t=4.975 df=74.96	

Corresponding to Fig. 1g

CON vs TCS

	"Object" (s)		"Central" (s)		"Stranger" (s)	
	Mean	SEM	Mean	SEM	Mean	SEM
CON	70.43	4.31	46.84	2.23	182.7	5.82
TCS	173.5	12.22	36.67	4.54	89.85	10.27

	Mean (s)	SEM (s)
CON	43.61	3.699
TCS	-29.47	8.23
95% confidence interval	-91.18 to -54.98	
Welch-corrected t, df	t=8.099 df=52.69	

Corresponding to Fig. 1h

Unpaired *t*-test with Welch's correction

	Mean (s)	SEM (s)
CON	4.368	0.55
TCS	16.9	2.43
95% confidence interval	7.492 to 17.57	
Welch-corrected t, df	t=5.02 df=41.9	

Supplementary information, Table S2

Table S2 Two-way repeated-measures analysis of variance (ANOVA) results (F statistics and *P* values for 'interaction effects' between chamber side and treatment)

Corresponding to Fig. 1f

CON vs TCS

	P value	*P* value summary	F
Interaction	< 0.0001	****	36.44
chamber	< 0.0001	****	89.72
treatment	0.911	ns	0.01253

Bonferroni post-hoc comparing "CON" vs "TCS" for each group

Chamber	Difference	t	Adjusted *P* value	Summary
Familiar	-69.04	6.727	< 0.0001	****
Central	15.32	1.492	0.411	ns
Stranger	51.73	5.041	< 0.0001	****

Corresponding to Fig. 1g

CON vs TCS

	P value	*P* value summary	F
Interaction	< 0.0001	****	85.7
chamber	< 0.0001	****	91.97
treatment	0.9999	ns	2.26E-08

Bonferroni post-hoc comparing "CON" vs "TCS" for each group

Chamber	Difference	t	Adjusted *P* value	Summary
Object	-103.1	9.699	< 0.0001	****
Central	10.17	0.9574	> 0.9999	ns
Stranger	92.89	8.742	< 0.0001	****
